# Physicochemical Modulation Strategies for Mass Production of Extracellular Vesicle

**DOI:** 10.1007/s13770-025-00726-9

**Published:** 2025-06-05

**Authors:** Hyoeun Park, Young-Kwon Seo, Yoshie Arai, Soo-Hong Lee

**Affiliations:** Department of Biomedical Engineering, Dongguk University, Seoul, South Korea

**Keywords:** Extracellular vesicle, Mass production, Chemical modulation, Mechanical modulation, 3D culture system, EV mimetic nanovesicles

## Abstract

*****BACKGROUND***:**

Extracellular vesicles (EVs) have attracted expanded attention as vehicles for the diagnosis and therapy of diseases and regenerative medicine due to their biocompatibility, efficient cellular uptake ability, and capacity to transport biologically active molecules. However, the low secretion yield of EVs and the challenges of large-scale production remain the main barriers to their extensive clinical use.

*****METHODS AND RESULTS***:**

This review explores recent strategies to enhance EV production in cell culture systems, focusing on chemical stimulation, mechanical stimulation, and structural stimulation. First, we review chemical stimulation strategies for modulating culture conditions using chemical stimulation, including nutrient composition, pH, temperature, oxygen levels, intracellular cholesterol, and oxidative stress. Second, we examine mechanical stimulation strategies, including shear stress, irradiation, and ultrasound. Third, we explore structural stimulation strategies, such as three-dimensional (3D) culture systems involving spheroid-based culture, as well as the use of bioreactors and scaffolds. In addition, cell-derived nanovesicles containing cell membrane and cellular component, which can be more easily mass-produced compared to EVs, are proposed as an alternative to EVs.

*****CONCLUSION***:**

Future research should focus on developing cost-effective and scalable EV production methods while improving purification techniques to ensure a high yield without compromising functional integrity. Moreover, integrating optimized stimulation strategies—such as refining 3D culture systems, bioreactor designs, and mechanical stimulation methods—could further enhance EV secretion. Addressing these challenges is essential for advancing EV-based applications in both research and clinical practice.

## Introduction

Extracellular vesicles (EVs) are lipid bilayer-enclosed nanoparticles secreted by all living cells [1]. They are present in almost all tissues and body fluids and serve as carriers of biological information derived from their parent cells, including lipids, proteins, cytokines, messenger RNA (mRNA), microRNAs (miRNAs), long noncoding RNAs (lncRNAs), and DNA fragments. EVs play essential roles in mediating intercellular communication and regulating various physiological processes [2]. Furthermore, they are actively involved in pathological environments, including immune responses [3], angiogenesis [4], aging [5], cancer [6], viral infections [7], endocrine disorders [8], and neurological diseases [9].

A variety of biosynthetic mechanisms for EVs have been characterized. EVs are primarily generated through the fusion of multivesicular bodies (MVBs) with the plasma membrane, a process orchestrated by various proteins, including the ESCRT complex, tetraspanins, syntenin, Rab proteins, and sphingomyelinase [10]. EVs can also form through plasma membrane budding, which is induced by calcium influx, cytoskeletal rearrangement, and the activity of enzymes such as floppies and scramblase [11]. During EV biosynthesis, molecules such as ATP, NADPH, GAPDH, Rab proteins, Rho GTPases, ROCK, and ARF6 play essential roles [12]. This process can vary depending on the cellular environment and external stimuli. EV cargo is concentrated through a stepwise process involving clustering, budding, fission, and vesicle release, after which the vesicles are delivered to recipient cells.

EVs contain lipids, nucleic acids, and proteins derived from their parent cells, and their composition varies depending on the cell type and environmental conditions. The nucleic acids carried by EVs reflect the genetic information of their originating cells and play key roles in regulating gene expression and mediating physiological responses. EVs also contain lipids such as ceramide, cholesterol, sphingomyelin, and phosphatidylserine, with ceramide being a critical component involved in EV biogenesis [13]. EVs can include membrane proteins, cytoplasmic proteins, extracellular matrix proteins, and serum proteins. Through the delivery of growth factors and signaling molecules, they contribute to processes such as cell growth and differentiation, immune regulation, and the control of cancer progression [14].

The bioactive molecules within EVs elicit functional responses in recipient cells upon their release into the extracellular space. These molecules can directly interact with target cell membranes, activating surface receptors and downstream signaling pathways. Additionally, EV membrane proteins facilitate adhesion, docking, and uptake, allowing EVs to enter recipient cells via clathrin-mediated or non-clathrin-mediated endocytosis [15]. Internalized EVs reach multivesicular bodies (MVBs) via the endosomal pathway, where they are either transported to lysosomes or release their cargo into the cytoplasm through back-fusion with MVBs [10]. The released EV cargo plays a crucial role in intercellular signaling and physiological responses by modulating various cellular functions. For example, EVs derived from embryonic stem cells contain heat shock protein 90 (HSP90), which upregulates Oct4 expression, promoting dedifferentiation in retinal cells and alleviating retinal degeneration (RD) [16]. Additionally, EVs from regulatory T cells (Tregs) carry immunosuppressive molecules such as TGF-β and IL-10, which help maintain immune tolerance and prevent autoimmune responses [16].

Given their unique biological properties, EVs have gained increasing attention in modern biomedicine, particularly in disease diagnosis, treatment, regenerative medicine, and drug delivery. Researchers are exploring the use of EVs as biomarkers for studying pathological conditions and facilitating early disease detection. For example, EVs extracted from body fluids can serve as diagnostic tools for diseases such as cancer [17], while their biological cargo can exhibit therapeutic effects. Notably, EVs derived from mesenchymal stem cells (MSCs) demonstrate therapeutic potential for conditions such as heart disease [18], diabetes [19], and neurological disorders [20], primarily due to the presence of nucleic acids such as miRNA and lncRNA. In addition, EVs hold promise in regenerative medicine applications, including skin regeneration [21] and bone-cartilage tissue repair [22]. Likewise, EVs secreted by immune cells can be utilized to improve cancer therapy efficacy or facilitate tissue regeneration [3]. Their nano-scale size allows for efficient cellular uptake, and as cell-derived materials, they exhibit high biocompatibility and low immunogenicity, making them ideal candidates for drug delivery. For instance, EVs originating from cancer cells can be engineered to carry cancer vaccines [23] or therapeutic agents [24], enhancing targeted cancer treatment.

Despite their remarkable potential, the limited production of EVs remains a significant technical barrier to their research and clinical applications. Since cultured cells secrete EVs in limited quantities, and the separation and purification processes are complex, time-consuming, and costly, scaling up EV production is a considerable challenge [25]. Addressing these limitations is crucial for advancing EV-based biomedical applications.

**Fig. 1:**
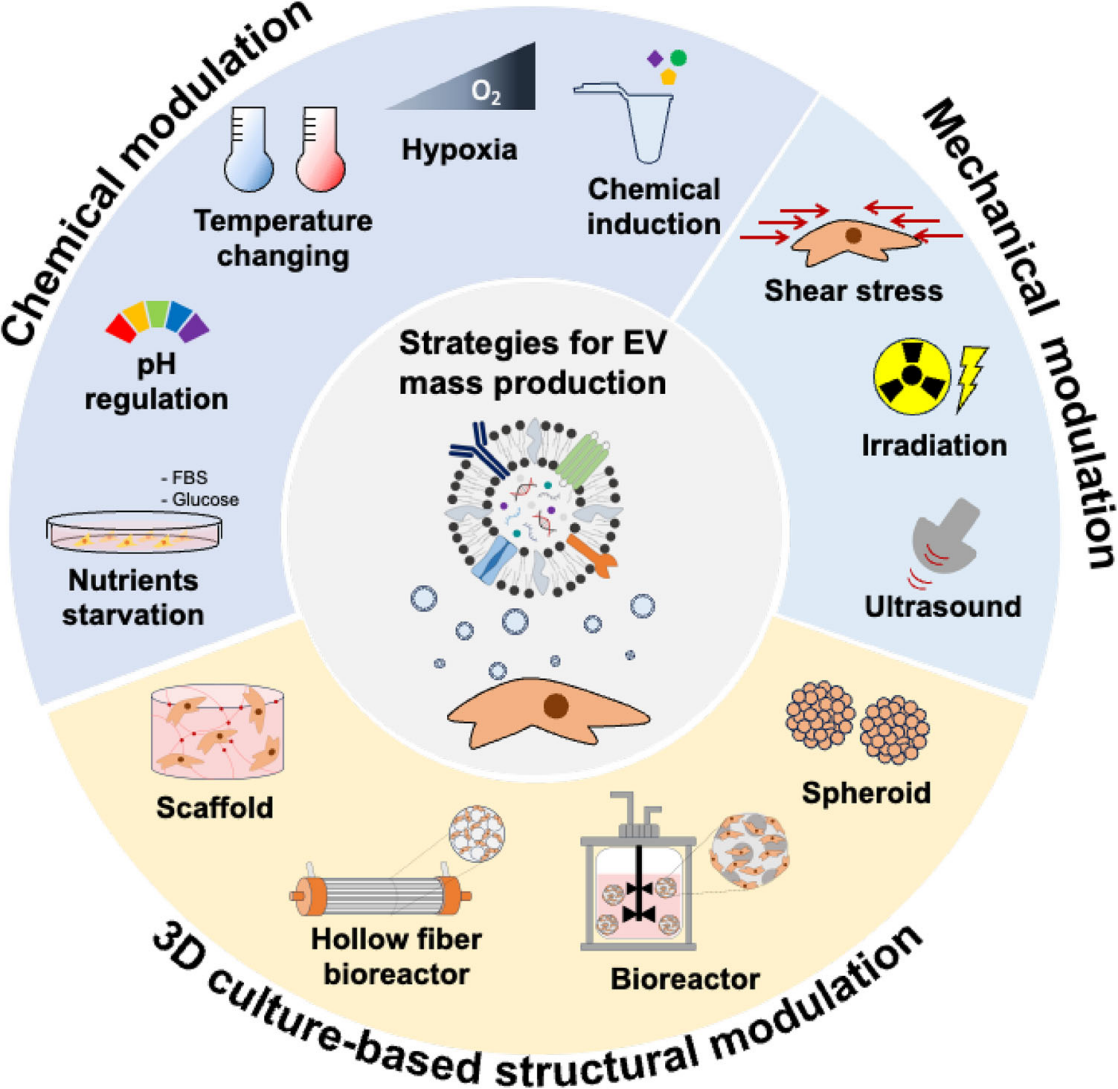
Strategies for the large-scale production of extracellular vesicles

This review discusses various strategies aimed at enhancing EV production in different cell culture systems. The first section explores chemical modulation methods to increase EV yield by modulating culture conditions, including nutrient composition, pH, temperature, oxygen levels, intracellular cholesterol, oxidative stress, and the use of cytokines or small molecules. The second section examines mechanical modulation methods, such as shear stress, irradiation, and ultrasound, as means of promoting EV secretion. The third section highlights structural modulation methods in cell culture, including 3D culture systems, such as spheroids-based culture, as well as the use of bioreactor and scaffolds, which facilitate large-scale cell growth and EV production. Lastly, we introduce an emerging strategy for EV mass production using cell-derived nanovesicles containing cell membranes and intracellular components. In this context, the terms used to describe EV yield strategies are categorized as follows. *High-efficiency production* refers to approaches that enhance EV output through chemical or mechanical modulation. *Large-scale production* involves the use of culture systems, such as 3D platforms or bioreactors, that enable the expansion of culture capacity and process scalability. *Mass production* is used as a comprehensive term encompassing both strategies. This classification is applied throughout the review to ensure clarity and consistency. By comprehensively reviewing these approaches, this study aims to propose novel solutions to the current challenge of low EV yield and expand their potential applications in both research and clinical fields (Fig. 1).

## Strategies for the large-scale production of extracellular vesicles

### Chemical modulation

The mass production of EVs has been investigated using different approaches, among which chemical approaches have become one of the most robust methods to increase EV production through the tunning of the intracellular signaling pathways and metabolic activities. Several chemical modifications within the cellular microenvironment can enhance EV secretion by regulating membrane fluidity, protein expression, vesicle biogenesis, and exocytosis. Approaches such as nutrient starvation, pH regulation, thermal modulation, hypoxia, cholesterol regulation, oxidative stress induction, and specific chemical treatments have been reported to promote EV production.

**Fig. 2:**
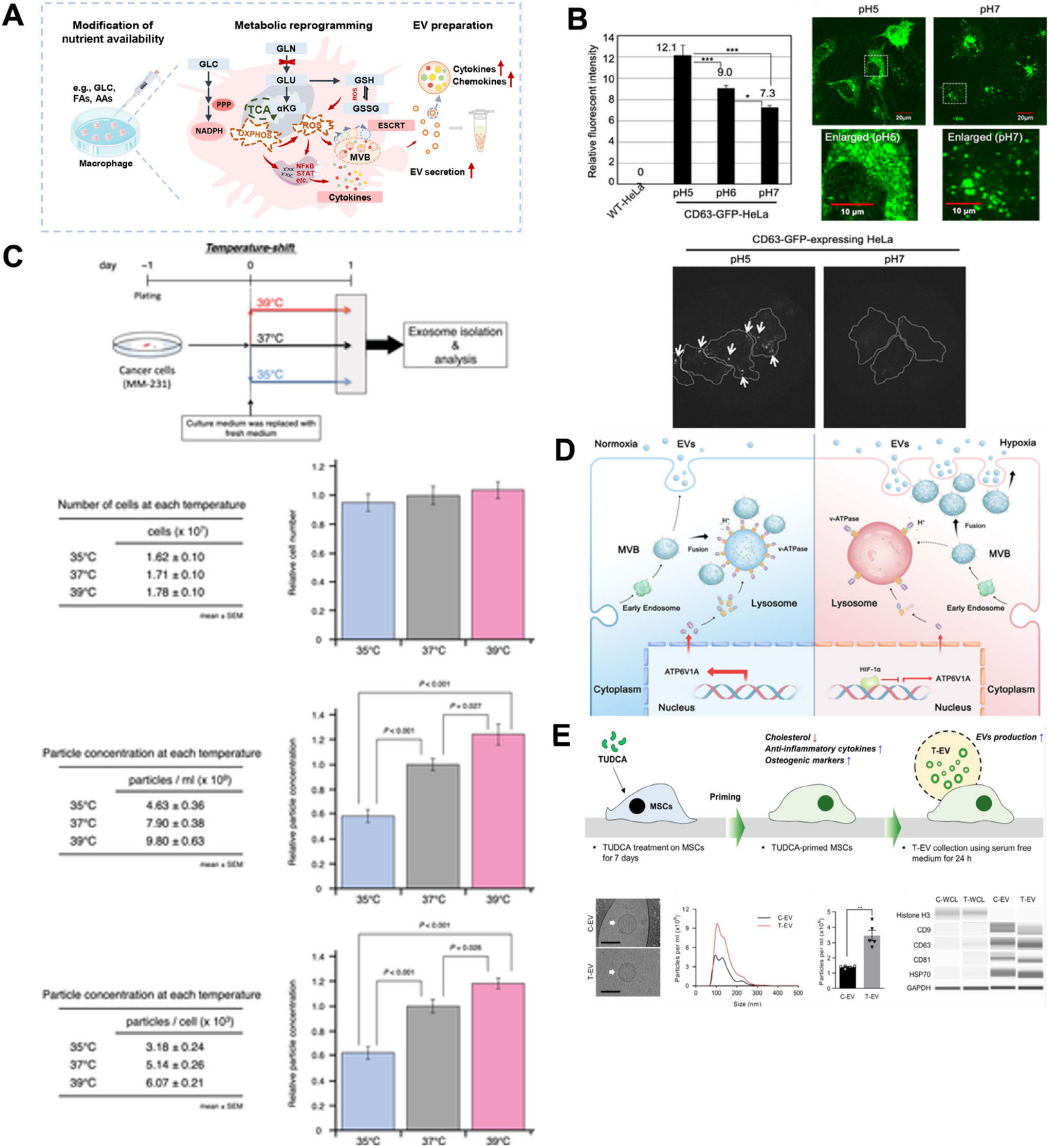
Chemical modulation strategies for increasing EV production. **A** Nutrients starvation. **B** pH regulation **C** Temperature change. **D** Hypoxia. **E** Cholesterol regulation. **A** adapted from Wang et al., CC BY-NC 4.0 [26], **B** adapted from Nakase et al., CC BY 4.0 [27], **C** adapted from Otsuka et al., CC BY 4.0 [28] **D** adapted from Wang et al., CC BY-NC-ND 4.0 [29] **E** reproduced from [30] with permission from Elsevier

Nutrient depletion increases the expression of EV biosynthesis-related genes (GTPase, Rab family, etc.) and induces metabolic stress, leading to increased EV secretion. pH regulation indicates that EV release is accelerated in acidic environments, with pH levels below 6.5 in various cancer cells shown to increase the observable content of EV production by up to 69-fold. Temperature changes can influence EV production by regulating MVB formation and cell membrane fusion, including an enhancement in EV release within 37–42 °C. Heat stress at 40–60 °C has also been reported to enhance EV secretion via induction of heat shock proteins and ATP release. Hypoxia activates the HIF signaling pathway, which contributes to enhanced EV release and increased Rab22A and Rab27a expression in breast and ovarian cancer cells. According to current research, cholesterol levels regulate EV release through the PI3K-Akt pathway, and oxidative stress has been shown to promote EV release by raising ROS levels, thereby activating the Caspase-3 pathway. In addition to these, norepinephrine, forskolin, and cisplatin have been observed to increase EV secretion by inducing ceramide generation and Rab27 protein expression. These chemical inducers offer highly scalable EV production methods, have broad applicability across cell types, and remain under investigation. In this section, general chemical regulatory strategies for improving EV production will be explained and classified into six categories (Fig. 2).

#### Nutrient starvation

The optimization of EV production depends on carefully regulating culture conditions and cellular metabolism. One of the most critical factors influencing EV yield is the composition of the culture medium, particularly the presence of fetal bovine serum (FBS) [31]. While FBS provides essential nutrients and growth factors, it can also introduce exogenous vesicles, ribonucleic acid contaminants, and protein complexes that interfere with endogenous vesicle production. These components can reduce EV purity and affect secretion dynamics. To address this, researchers have investigated the effects of serum depletion on EV production. And several reports reducing or removing serum from the culture medium tends to increase in EV secretion. Li et al. demonstrated that N2a cells cultured under serum-free conditions for 120 h produced a higher EV yield without significant changes in vesicle size or characteristics. This increase was associated with the upregulation of genes linked to EV biogenesis and secretion, including G-protein, GTPase, and Rab family genes. Among them, ARF6 was the most upregulated, suggesting that its role in multivesicular body (MVB) docking contributed to enhanced EV release [32]. Similarly, Bost et al. reported that HEK293T cells cultured in serum-free Opti-MEM showed a rise in EV secretion, accompanied by changes in sphingomyelin-related pathways. interestingly, this effect was observed regardless of the tetraspanin-based engineering approach used, suggesting that Opti-MEM promotes EV production independently of cell type [31]. Also, Sun L. et al. demonstrated that reducing FBS concentration to 1% induced metabolic reprogramming in multiple myeloma cell lines (RPMI 8226, U266, KM3), leading to a 2.5- to 4.3-fold increase in EV production [33]. However, not all studies report the same effect. Saludas L. et al. found that serum starvation significantly reduced EV secretion in cardiac progenitor cells. Moreover, when serum-depleted EVs were added back to the culture medium, it led to a higher EV yield compared to serum-free conditions [34]. This suggests that certain proteins and EVs present in serum may contribute to the overall EV production.

In addition, glucose starvation has also been shown to enhance EV secretion by inducing metabolic stress. Garcia et al. observed that cardiomyocytes (H9C2 cells) cultured in glucose-free conditions for 48 h exhibited a fourfold increase in EV release [35]. Similarly, Bae et al. demonstrated that mTORC1 inhibition in TSC-null cells increased EV secretion under glucose deficiency [36]. These findings suggest that metabolic stressors such as nutrient deprivation can regulate EV release through intracellular signaling pathways. Furthermore, Wang et al. reported that M1 macrophages (M1-Mφ) released three times more EVs under glutamine-depleted conditions. This increase appeared to be driven by shifts in energy metabolism and redox balance, particularly an increase in reactive oxygen species (ROS) and the activation of pathways related to EV biogenesis and cargo sorting, such as the ESCRT [26]. These studies highlight the importance of optimizing culture conditions to enhance EV production. Given the variability in cellular responses to serum and nutrient deprivation, further optimization and systematic evaluation are necessary to establish standardized conditions that increase EV yield while maintaining vesicle integrity and functionality. However, despite these promising results, nutrient starvation strategies have inherent limitations. Excessive nutrient deprivation can compromise cell viability, alter cellular behavior, and potentially impair the functionality of secreted EVs. In addition, if not carefully controlled, such conditions may lead to elevated oxidative stress, mitochondrial dysfunction, or other undesirable cellular responses. Therefore, while metabolic stress can enhance EV secretion, it must be precisely regulated to avoid compromising EV quality or overall cellular health.

#### pH modification

In cell culture, maintaining optimal pH is essential for cell survival, proliferation, and EV production. The commonly used pH range of 7.2 to 7.4 closely mimics the physiological conditions of human blood (pH 7.35–7.45), providing a natural environment for cells to thrive. This range ensures the stability of enzymes, proteins, and other critical components in the culture medium, optimizing metabolic processes and cellular functions. Acidic environments, such as those found in tumor microenvironments, have been associated with enhanced EV release. Recent studies have investigated the role of pH in EV production, revealing that lower pH conditions significantly increase EV yield. A different external factor that triggers cell EV production is alterations in proton levels. Cancer cells, in particular, modify the pH of their microenvironment to enhance EV release. For instance, Parolini I. et al. demonstrated that melanoma cells (Mel1) released a higher quantity of EVs in an acidic environment compared to a buffered one [37]. Similarly, Ban et al. observed that culturing HEK 293 cells at pH 4, pH 7, and pH 11 resulted in increased EV production as the pH decreased. Specifically, under acidic conditions, the concentration of exosomal proteins and RNA was six times higher compared to neutral conditions [38]. Nakase et al. reported a similar finding with A431 and HeLa cells, noting a 2.5-fold increase in EV secretion at pH 5 compared to pH 7. Logozzi et al. expanded this research by analyzing the relationship between pH and EV production in several human cancer cell lines, including LNCaP, Me30966, SaOS2, SKBR3, and HCT116 [39]. To mimic the acidic tumor microenvironment, these cells were gradually adapted to acidic pH conditions. As the microenvironmental pH decreased from 7.4 to 6.5, a dramatic increase in EV release was observed, with a 48- to 69-fold increase in SKBR3, Me30966, and HCT116 cells, and a 9- to 22-fold increase in LNCaP and SaOS2 cells. In contrast, alkalinization of the cells suppressed EV release, as evidenced by a gradual decline in EV secretion when the pH was shifted from 6.5 to 7.4, further confirming the pH dependency of EV production. Researchers hypothesize that low pH conditions may promote EV release as a mechanism to prevent the accumulation of toxic substances within cells. However, the precise mechanisms by which pH affects EV production and changes in EV composition remain unclear. In acidic environments, the EV membrane shows increased levels of sphingomyelin (SM) and GM3 lipids, which enhance membrane rigidity and regulate fusion efficiency [40]. Additionally, proton pumps are activated in low pH conditions, facilitating EV release. When proton pump inhibitors (alkalinizing agents) are applied, EV release is significantly reduced, further supporting the role of proton pump activity in the process.

The research on the relationship between low pH and EV release is limited in cancer studies. However, considering that pH regulation affects gene expression, metabolic activity, and stem cell differentiation in normal cells, it can be inferred that pH may also regulate EV secretion in other cell types. Additionally, pH could potentially induce functional changes in EVs, highlighting the need for further in-depth studies.

#### Temperature change

Temperature plays a crucial role in the physiology, development, and behavior of living organisms. In humans, tissue and body temperature are tightly regulated under normal conditions, but during disease states, this regulation can become disrupted. For example, depression is often associated with elevated body temperature, and tumors such as lymphomas and breast cancer exhibit higher temperatures at the tumor site compared to normal tissues, with differences reaching up to 3.5 °C. These temperature variations create a microenvironment that can influence cellular processes, including EV secretion. Studies have shown that temperature modulation has a significant impact on EV production, making it a promising strategy for high-efficiency EV biomanufacturing. One of the most critical steps in EV biogenesis is the formation of multivesicular bodies (MVBs) and their fusion with the plasma membrane. This process serves as a key regulatory checkpoint for EV release. Mahmood et al. demonstrated that temperature directly affects MVB docking and fusion kinetics [41]. Their study revealed that higher temperatures, such as 37 °C, increase the frequency of MVB fusion and the dynamics of EV release, while lower temperatures, such as 23 °C, reduce these rates. This finding highlights the potential of temperature as a controllable factor to enhance EV secretion. Thermal stress has also been shown to significantly enhance EV secretion. For instance, Hedlund et al. when Jurkat (T cell leukemia) and Raji (B cell leukemia/lymphoma) cells were exposed to thermal stress at 40 °C for 1 h, EV production increased by 3- and 22-fold, respectively [42]. Similarly, Chen et al. reported that 3LL lung cancer cells subjected to 42 °C for 1 h, followed by a 4-h recovery period, exhibited a 3.5-fold increase in EV secretion compared to cells cultured under normothermic conditions [43]. This enhancement was attributed to heat stress-induced ATP release and calcium influx, both of which are key regulators of vesicular transport and EV release. Yang et al. further demonstrated that thermal shock (~ 60 °C) elevates heat shock protein (HSP) expression, promoting nanochannel formation on the plasma membrane and enhancing EV release. Additionally, HSP-mediated p53 activation has been linked to increased EV production via the TSAP6 mechanism [44]. Incremental changes in temperature have also been investigated to evaluate their effects on EV secretion. Otsuka et al. found that the thermostable low-density lipoprotein receptor (LDLR) is upregulated in breast cancer cells under hyperthermic conditions, contributing to temperature-dependent EV secretion. A comparative analysis at 35–39 °C showed higher temperatures correlated with increased EV production [27]. Xu et al. observed that exposing MCF-7/ADR breast cancer cells to 45 °C resulted in a twofold increase in EV production, which was linked to the upregulation of Rab7b, a gene involved in EV biogenesis [45]. Similarly, Barekzai et al., MSCs exhibited the highest EV production rates at 35 °C and 38 °C, highlighting the importance of temperature in optimizing EV yields [46].

Temperature modulation is promising to enhance EV production, but several limitations remain. Elevated temperatures can induce cellular stress, reduce cell viability, and alter cellular states, potentially compromising EV quality. Thermal stress may also affect EV cargo composition, leading to functional variability that may limit clinical applications. In addition, the molecular mechanisms behind temperature-induced EV secretion—such as ATP release, calcium influx, HSP expression, and the roles of Rab7b and LDLR—are not yet fully understood. Extreme temperatures may further reduce scalability and reproducibility across cell types. Therefore, while temperature optimization holds potential for large-scale EV production, careful calibration is required to maintain cell health and EV functionality.

#### Hypoxia

Hypoxia is a condition of insufficient oxygenation in cells and tissues that plays a physiologically significant role in cancer, tissue regeneration, and immune responses by modulating cellular and tissue adaptations to low oxygen levels [47]. It is commonly observed in tumor tissues. Normal tissues generally have oxygen pressures ranging from 40 to 60 mmHg, while oxygen levels in tumor tissues are markedly lower, around 10 mmHg [47, 48]. The hypoxia environment in tumors is primarily attributed to the high oxygen needs of aggressively proliferating cancer cells and the poor oxygen supply due to the distance between tumor tissue and blood vessels. Hypoxia within tumor tissues promotes metastasis, chemoresistance, and immune escape mechanisms. In tissue regeneration, hypoxia plays a key factor in regulating stem cell differentiation, particularly in cartilage and bone regeneration. Hypoxia-inducible factors (HIFs), transcription factors activated under hypoxic conditions, mediate cellular responses to oxygen depletion by regulating gene expressions for angiogenesis, metabolic reprogramming, and the epithelial-to-mesenchymal transition (EMT). Recent research has found that hypoxia stimulates the EVs production. In hypoxia environments, the plasma membrane receptor expression is changed, leading to increased receptor activation, internalization, or receptor clustering. These processes enhance endocytosis and promote EV release, contributing to the functional and quantitative dynamics of EV production under hypoxia. In a study by King H.W. et al. breast cancer cells exhibited a significant increase in EV secretion under conditions of moderate and severe hypoxia (1% and 0.1% O_2_, respectively) [49]. Wang et al., under hypoxic conditions (1% O_2_ for 24 h), human breast cancer cell lines (MCF-7, MDA-MB-231, and MDA-MB-435) showed a significant increase in EV production compared to normal conditions (20% O_2_). This effect was mediated through a HIF HIF-dependent increase in RAB22A expression, which induced EV budding at the plasma membrane [50]. Similarly, ovarian cancer cells exposed to hypoxia demonstrated enhanced EV production through the activation of Rab27a, suppression of Rab7, LAMP1/2, and NEU-1, and the induction of a more secretory lysosomal phenotype. In addition, Nonetheless, hypoxia appears to also influence the composition of EVs, as evidenced by the upregulation of various bioactive factors involved in angiogenesis in endothelial progenitor cell-derived EVs. Most of these investigations have centered on cancer cell lines, known for their heightened EV secretion capacity. Also, Hypoxic preconditioning increased the EV secretion of UC-MSCs. Liu et al. cultured hypoxic preconditioning UC-MSCs in a 1% O_2_ incubator, and they showed that the concentration of EVs in the hypoxic preconditioning group was significantly higher than in the normoxia group (about 1.5-fold). Hence, further validation and optimization of these findings are necessary in non-cancerous cell models. Moreover, it's crucial to recognize that subjecting cells to stressful culture conditions may impact the characteristics, composition, and functions of the resulting EVs. Additionally, stress-induced cell death may elevate the presence of apoptotic bodies, potentially leading to inaccuracies in EV yield assessments. Therefore, comprehensive evaluations of EV yield, quality, and functionality are imperative.

#### Cholesterol regulator

Cholesterol is a major component of the cell membrane and plays a critical role in regulating membrane fluidity, signal transduction, and focal adhesion [51]. Changes in membrane cholesterol levels affect protein cluster assembly, as well as key biophysical properties such as the permeability of polar molecules and bending modulus [52]. Several studies have demonstrated that cholesterol suppression enhances EV production. Abdullah et al. reported that ß-cyclodextrin treatment (~ 2 mM) in astrocytes reduced cholesterol levels and significantly increased EV release. Furthermore, cholesterol depletion was shown to regulate EV production through downregulation of the PI3K-Akt pathway, indicating cholesterol-dependent EV production at both the cellular level and *in vivo* using mice [53]. In another study, Cha et al., 2.4 mM of Tauroursodeoxycholic acid (TUDCA)-treated MSCs for 7 days produced significantly more EVs than conventional MSCs. TUDCA is an amphiphilic small molecule that reduces intracellular cholesterol levels [54]. TUDCA-induced intracellular cholesterol reduction in MSCs upregulated the expression of caveolin-1 (CAV1) and lysosomal-associated membrane protein 1 (LAMP-1), key mediators of exocytosis, leading to an increase in EV production [30]. In addition, Martin et al. demonstrated that pharmacological and genetic inhibition of cholesterol biosynthesis in various cell types resulted in a nine-fold increase in EV yield. In HEK293 cancer cell lines, siRNA-mediated suppression of cholesterol synthesis led to a 14 to 26-fold increase in EV production. Similarly, MSCs treatment with 5 nM statins as known cholesterol synthesis inhibitors decreased intracellular cholesterol levels and enhanced EV secretion by 4.7–sixfold [55]. In contrast, EV membranes are known to be rich in cholesterol, and elevated intracellular cholesterol levels have also been reported to promote EV biogenesis and enhance EV production [56, 57]. Guix et al. observed a massive release of EVs in aged neurons in mice, identifying accumulation as the underlying cause. Furthermore, treatment of N2A neuronal cells with U18666A led to cholesterol accumulation and a twofold increase in EV production [58]. In addition, Chen et al. reported that SKOV-3 (Ovarian cancer cell line) treated with statin decreases cholesterol synthesis and EV secretion by inhibiting HMG-CoA reductase [59]. Notably, external cholesterol sources such as oxidized LDL (OxLDL) have been shown to enhance EV release. Roldán Gallardo et al. demonstrated that treating human prostatic stromal cells (HPSCs) with OxLDL significantly increased the release of EVs [60]. These contradictory findings suggest a complex relationship between cholesterol regulation and EV production. Therefore, further research is needed to elucidate the fundamental mechanisms underlying cholesterol-mediated EV production and to compare the effects of cholesterol modulation and EV production across different cell types, ultimately leading to the establishment of standardized protocols. Nevertheless, cholesterol modulators offer a cost-effective and scalable approach for enhancing EV yield, making them highly promising for GMP-compliant EV mass production and contributing significantly to the development of EV-based therapeutics. However, both cholesterol depletion and accumulation have been reported to enhance EV release, depending on the cell type and experimental context. This bidirectional effect highlights the need for precise control and a deeper mechanistic understanding to avoid unintended alterations in EV composition or function.

#### Oxidative stress inducer

Oxidative stress refers to cellular damage caused by excessive accumulation of reactive oxygen species (ROS) generated during intracellular oxygen metabolism [61]. ROS levels can also increase due to external stimuli such as UV radiation, ionizing radiation, heavy metals, and chemical substances. High concentrations of H_2_O_2_ are well known to increase ROS levels, leading to oxidative stress-induced cellular dysfunction or apoptosis [62]. Moreover, oxidative stress has been linked to increased EV production. Yang et al. reported a 25% increase in EV production in HEK293 cells upon treatment with 25 µM H_2_O_2_, which was accompanied by elevated oxidative stress [63]. In another study, et al. found that treating Jurkat T-cell leukemia cells with 100 µM H_2_O_2_ and Raji B-cell leukemia/lymphoma cells with 50 µM H_2_O_2_ resulted in a 15-fold and 32-fold increase in EV release, respectively, due to oxidative stress [42]. Additionally, Benedikter et al. reported an increase in EV release from BEAS-2B human bronchial epithelial cells treated with 15 µM acrolein, further supporting the role of oxidative stress in EV production [64]. These studies suggest that chemical stimuli and oxidative stress enhance EV production by modulating cellular signaling and metabolic pathways, providing valuable insights for the development of EV-based therapeutics and industrial-scale EV production.

Similarly, Verma et al. reported that exposure of CYP2E1-expressing HepG2 liver cancer cells to ethanol resulted in a 1.9- and 3.3-fold increase in EV yield with 50 mM and 100 mM ethanol, respectively. This increase was found to be mediated through the activation of the Caspase-3 pathway, which regulates the actin-based machinery required for EV exocytosis via ROCK-1 signaling [65]. Likewise, Malik et al. showed a significant increase in EV production in rat cardiomyocytes after just 2 h of ethanol exposure, with reactive oxygen species (ROS) playing a central role in this process [66]. Additionally, Bala et al. observed an increase in Rab family protein expression following ethanol treatment, further implicating this pathway in the regulation of EV release [67]. The increase in EV release under oxidative stress is believed to function as a cellular defense mechanism that exports damaged molecules from the cell, potentially accompanied by stress response proteins. Additionally, excessive oxidative stress induces apoptosis and releases apoptotic bodies thereby contributing to increased EV production. Therefore, it is essential to establish standardized oxidative stress conditions and analyze the content change in EV. Since molecular changes in EVs can alter their functional properties, further research is needed to optimize EV production through oxidative stress modulation according to therapeutic objectives1. However, excessive oxidative stress can cause cytotoxicity, induce apoptosis, and lead to the release of EVs with altered or inconsistent cargo. These effects may reduce the safety and functional reliability of EVs, especially for therapeutic use. Therefore, precise control of oxidative stress conditions is essential to ensure EV quality and application potential.

#### Chemical compound

Additionally, chemical compound induction has been shown to increase EV release by directly affecting cellular responses, in contrast to the modulation of the cellular microenvironment. Xiao et al. observed a fivefold increase in EV production when A549 lung adenocarcinoma cells were treated with the DNA-damaging chemotherapy agent Cisplatin (DDP) at 3 µg/mL for 24 h [68]. This increase was attributed to the release of cisplatin through EVs, serving as a survival mechanism for the cells.

Furthermore, recent research by Wang et al. demonstrated that various compounds can stimulate EV secretion from MSC. They found that 50 µM Fenoterol increased EV production by approximately 1.7-fold, while Norepinephrine (NE), Forskolin (FK), N-Methyldopamine (MeDA), and Mephenesin (Mepn) significantly enhanced EV production in a dose-dependent manner. Notably, when Norepinephrine and N-Methyldopamine were co-treated, a threefold increase in EV production was observed, demonstrating a synergistic effect [69]. These compounds activate cellular metabolic pathways and increase the expression of genes involved in EV secretion, such as nSMase2, a key regulator of ceramide production and MVB regulation [70], as well as Rab27a [71] and Rab27b [72], which are essential for EV trafficking. MVB is an intracellular organelle containing small vesicles that can be released as EVs. It plays a key role in regulating EV formation.

In addition to chemical induction, another strategy for enhancing EV production is genetic modification. Kojima et al. developed a method to boost EV production by genetically encoding genes related to EV biogenesis. They identified and combined genes such as STEAP3, L-aspartate oxidase, and syndecan-4. These genes are associated with key processes like EV biogenesis, activation of the citric acid cycle, and formation of MVBs. When each gene was introduced into EV-producing cells, EV production increased significantly. The combination of all three genes resulted in a dramatic increase in EV yield. HEK-293T cells showed a 40-fold increase, and hMSCs showed a 15-fold increase [73]. This demonstrates the potential of genetic modification as a powerful tool for enhancing EV production, offering a scalable method for high-efficiency production and therapeutic applications. However, both chemical stimulation and genetic modification may raise concerns regarding cell toxicity, long-term stability, and safety, particularly in therapeutic applications. Careful evaluation and optimization are required to ensure consistent EV quality and minimize potential risks.

### Mechanical modulation

**Fig. 3:**
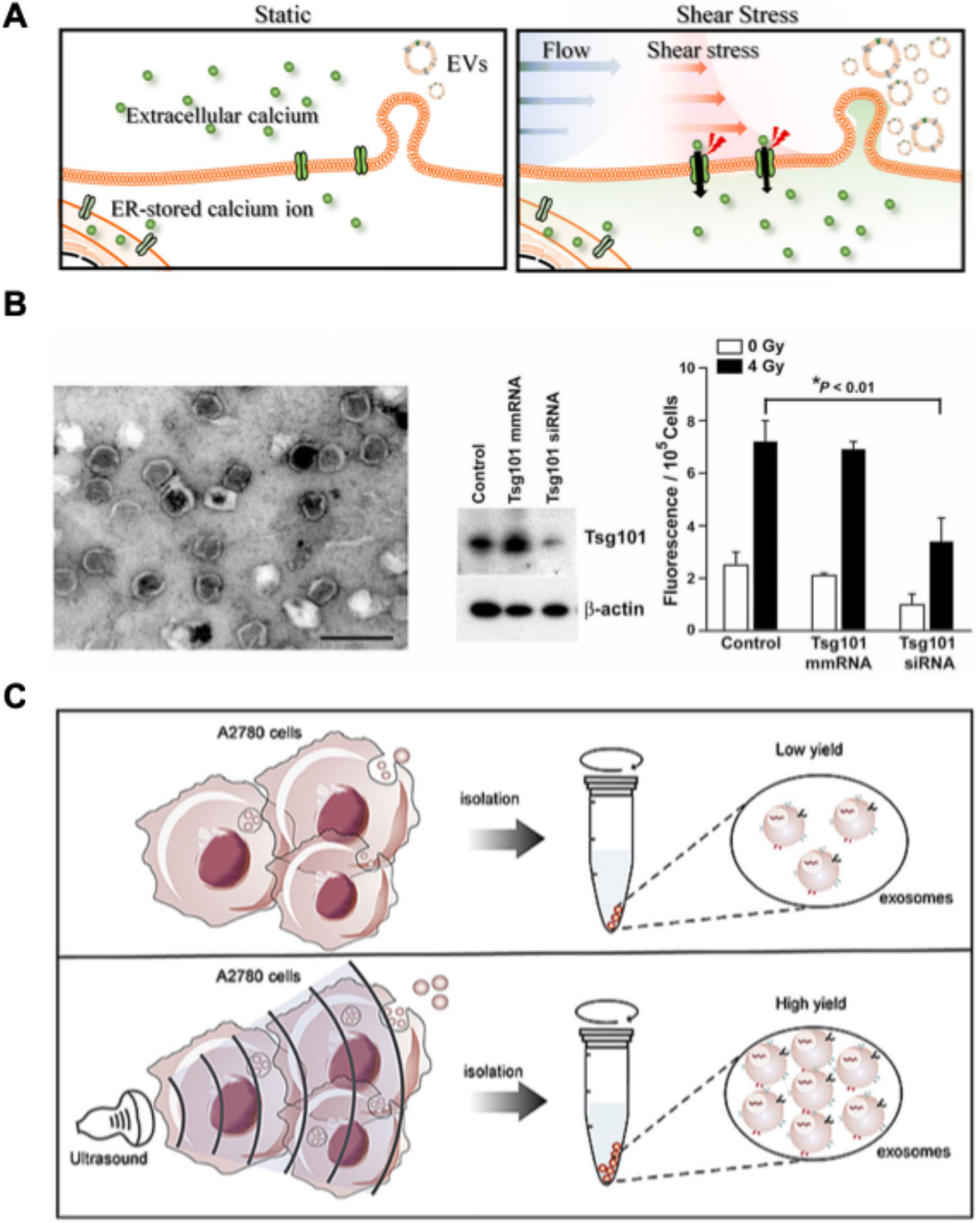
Mechanical modulation strategies for EV mass production. **A** Shear stress induces EV production through calcium influx. **B** Irradiation exposure increases EV secretion. **C** LIUS stimulation enhances EV yield in A2780 cells. **A** Reproduced from [74] with permission from The Royal Society of Chemistry. **B** reproduced from [75] with permission from American Association for Cancer Research. **C** reproduced from [76] with permission from Elsevier

Mechanical stimulation has emerged as a potent approach to improve EV production by leveraging the inherent mechanosensitivity of cells to physical forces. Many mechanical stimulation (i.e., shear stress, irradiation, and ultrasound stimulation) could modulate membrane dynamics, cytoskeletal organization, and intracellular signaling pathways, increasing EV secretion. This approach exploits the ability of cells to sense and adapt to mechanical forces to improve EV yield for industrial applications in disease treatment and diagnosis. Fluid flow-induced shear stress can promote EV release through mechanotransduction mechanisms like intracellular calcium influx and upregulation of signaling pathways contributing to EV biogenesis. Radiation is known to cause DNA damage and cellular stress responses producing a compensatory increase in EV production. damaged DNA, miRNA, and stress-related molecules are rich in EVs secreted from irradiated cells that contribute to regulating tumor microenvironments and encouraging radioresistance. Upregulation of signal pathways in response to radiation has been identified as a key mechanism driving EV biogenesis. Ultrasound stimulation offers a non-invasive and scalable method for EV production that utilizes acoustic cavitation, increased membrane permeability, and enhanced calcium influx to stimulate EV release. Low-intensity pulsed ultrasound (LIPUS) is shown to upregulate EV-related gene expression and cytoskeletal activity, and high-intensity ultrasound promotes EV release through it mechanical and thermal effects. Ultrasound-based technologies are adaptable and accessible and offer great potential for high-efficiency EV biomanufacturing. By fine-tuning stimulation parameters such as shear intensity, radiation dose, ultrasound frequency, and exposure duration, mechanical stimulation provides an effective means to augment EV production. This section summarizes the mechanisms of EV bioprocessing and mass production by shear stress, radiation, and ultrasound stimulation (Fig. 3).

#### Shear stress

Shear stress is a physical force generated by the flow of fluid across a surface [77]. *In vivo*, cells are exposed to shear stress through the movement of blood, lymph, or synovial fluid. This mechanical force directly affects cells by altering their morphology, cytoskeletal structure, ion channel activity, and gene and protein expression. *In vitro*, shear stress can be induced during cell culture using devices such as microfluidic systems, bioreactors, orbital shakers, or pressure-regulating instruments to analyze its effects on cellular behavior and function. These responses to shear stress can lead to phenotypic changes, including increased EV secretion, demonstrating the impact of mechanical stimuli on cellular behavior. Sangha et al. observed a 6.6-fold increase in EV production from RBCs exposed to shear stress. They discovered that Piezo1, a calcium ion channel, was activated by shear stress. This activation facilitated the influx of Ca^2+^ into cells and enhanced EV release. RBC-EVs produced under shear stress conditions showed higher purity than those generated with calcium ionophore treatment. This finding reflects the physiological relevance of these conditions, as RBCs are naturally exposed to it within the vasculature [78].

Seo et al. investigated the effect of shear stress on hBM-MSCs using a shaking culture system. Cells were exposed to shear stress by shaking at 50 rpm for one day. The presence of extracellular Ca^2+^ significantly influenced EV production under shear stress. With Ca^2+^ present, EV yield increased by fivefold, whereas in the absence of Ca^2+^, a smaller increase of 1.84-fold was observed. Although the EV production varied depending on the Ca^2+^ availability, the increase in EV yield under calcium-free conditions suggests that shear stress does not solely rely on Ca^2+^ to enhance EV release. Notably, no changes in ROS levels were detected under shear stress conditions. Based on these findings, the authors hypothesized that shear stress promotes EV release through caspase-independent signaling pathways [74]. The effect of shear stress on EV production has also been observed *in vivo*. Gao et al. reported that interstitial flow generates baseline shear stress ranging from 0.06 to 0.6 dyn/cm^2^ in healthy arteries. In atherosclerotic arteries, interstitial flow shear stress increases to 0.33 to 3.27 dyn/cm^2^, resulting in a twofold increase in EV production. This increase is mediated by EGFR receptor stimulation, which alters KLF5 expression. These changes influence vascular smooth muscle cell (VSMC) phenotypic switching and EV release [79]. These findings highlight the dynamic role of shear stress in regulating EV production under both physiological and pathological conditions. Shear stress has also been shown to enhance EV production in cancer cells. Wang et al. exposed human cervical squamous carcinoma (HeLa) and human malignant breast cancer (MDA-MB-231) cell lines to shear stress (10 dyn/cm^2^) using an orbital shaker for 60 min. The EV production in these cells increased twofold, which the authors attributed to a homeostatic response to mechanical stress [80]. Furthermore, acute shear stress in tumor cells induced autophagosome accumulation and fusion with MVBs, forming AP-MVB compartments. These compartments released EVs containing autophagy-associated proteins into the extracellular space, which alleviated cellular stress and provided a protective mechanism for tumor cells under mechanical stress.

However, not all cells show increased EV production under shear stress. Takafuji et al. reported no significant change in EV production in mouse C2C12 cells exposed to fluid flow shear stress at 250 rpm and 6 dyn/cm^2^ using an orbital shaker [81]. Similarly, Vion et al. found that high shear stress at 20 dyn/cm^2^ inhibited EV release in HUVECs. In contrast, low shear stress at 0.2 dyn/cm^2^ increased EV secretion by 2.5- to threefold through cytoskeletal remodeling [82]. These findings demonstrate the variability in cellular responses to shear stress and highlight the need to optimize shear stress conditions for each cell type.

In summary, shear stress is a powerful tool for enhancing EV production by leveraging the natural responsiveness of cells to mechanical stimuli. Studies have shown that shear stress promotes EV release by activating pathways such as Piezo1-mediated calcium influx, cytoskeletal remodeling, and caspase-independent signaling. However, the extent of EV production varies depending on factors like cell type, shear stress intensity, and the presence of calcium ions. While most studies indicate that shear stress promotes EV release, others show limited or no response under some conditions. Optimizing shear stress parameters, including magnitude, duration, and environmental factors, is essential for achieving maximizing EV production. This approach holds promise for addressing the challenges of high-efficiency EV mass production and expanding their applications in therapeutic and diagnostic fields.

#### Irradiation

Radiation exerts its cytotoxic effects primarily through the induction of DNA damage, ultimately leading to cell death [83]. Due to this property, radiation is widely used in cancer therapy for localized tumor control, symptom alleviation, and inhibition of cancer progression. Following radiation treatment, the disruption of homeostasis within the tumor microenvironment triggers a compensatory response by cells, resulting in a significantly increased EV secretion. Studies have consistently demonstrated that irradiated cells release a higher quantity of EVs compared to non-irradiated cells. Importantly, these EVs carry various biomolecules, including damaged DNA, which are transported to the extracellular space. This process may potentially alleviate intracellular damage, reduce radiation-induced cell death, and enhance cellular resistance to radiation.

For example, Amrita K. Cheema et al. observed a significant increase in EV concentration in plasma of irradiated nonhuman primates (NHPs) on the first and fourteenth days after exposure to 5.8 Gy (LD20-40/60) of total-body γ-radiation [84]. Similarly, Nasrollah Jabbari et al. reported a dose-dependent increase in EV biogenesis and secretion in MCF-7 human breast cancer cells 48 h after exposure to 2–10 Gy of radiation. They identified TSAP6 and Rab11 as key regulators of promoting EV biogenesis and secretion in irradiated MCF-7 cells [85]. Jella et al., demonstrated that γ-rays irradiation (0.005–0.5 Gy) in HaCaT human keratinocytes for 1 h enhanced EV release which was associated with increased ROS production and calcium influx [86]. Furthermore, Lehmann et al. found that irradiation-induced senescence in the 22Rv1 human prostate carcinoma cell line, following exposure to 4 Gy of radiation, led to a threefold increase in EV production mediated by a p53-dependent mechanism [75].

Radiation exposure not only affects directly irradiated cells but also induces radiation-induced bystander effects (RIBE), where signaling from irradiated cells influences neighboring non-irradiated cells. This includes alterations in exosomal cargo, such as miRNAs, which can either induce cellular damage and apoptosis or enhance radiation resistance.

Radiation-induced high-efficiency EV production offers a promising strategy for leveraging these EVs as effective tumor therapeutics. However, to fully realize this potential, further research is required to elucidate the molecular composition within EVs derived from irradiated cells. Additionally, optimizing parameters such as radiation intensity, exposure duration, and related conditions will be critical to achieving consistent and scalable production of therapeutic EV. Beyond cancer therapy, investigating the effects of radiation on EV release in non-carcinoma cells and analyzing their altered molecular characteristics could uncover new applications in regenerative medicine, immunology, and other fields.

#### Ultrasound stimulation

Ultrasound (US) refers to sound waves with frequencies exceeding the upper limit of human hearing, typically above 20 kHz [87]. These high-frequency mechanical vibrations can penetrate various materials or biological tissues, inducing mechanical, thermal, and chemical effects through physical interactions. This property makes ultrasound a versatile tool for applications in medical diagnostics, therapeutic interventions, and biological research [88]. Ultrasound stimulation has been widely utilized in therapeutic applications due to its physiological effects, including cell proliferation, suppression of inflammatory signaling, and enhanced cell membrane permeability [89]. These effects support its use in tissue regeneration, drug delivery, and increasing the bioavailability of therapeutic agents [90]. Ultrasound achieves these outcomes through mechanisms such as acoustic cavitation, thermal effects, and acoustic streaming, which directly influence cellular behavior and the surrounding microenvironment. In the field of EV research, ultrasound-mediated stimulation plays a pivotal role in enhancing EV production and functional modification. Ultrasound is a valuable tool in EV engineering, facilitating drug loading into extracted EVs and improving EV uptake by target cells or tissues. Recent studies have highlighted the potential of ultrasound stimulation for the large-scale production of EVs. Ultrasound enhances cell membrane permeability, enabling the uptake of desired intracellular molecules and significantly increasing EV secretion. Moreover, ultrasound and microbubble (USMB)-assisted techniques have shown high efficacy in promoting EV release. Ultrasound technology is highly accessible and cost-effective, as it can be implemented using standard laboratory equipment. The development of advanced ultrasound transducers with multi-channel systems enables high-efficiency and high-throughput EV production, making it a viable option for scalable manufacturing. By integrating ultrasound technology into EV bioprocessing workflows, researchers can achieve consistent and high-quality EV production without compromising functionality. Given its non-invasive nature, safety profile, and ability to enhance EV production, ultrasound-mediated stimulation represents a promising strategy for high-efficiency EV manufacturing. This combination of efficiency, accessibility, and versatility positions ultrasound as a transformative tool for advancing EV-based therapies and research applications.

Ultrasound can be classified into low-intensity pulsed ultrasound (LIPUS) and high-intensity ultrasound based on its intensity. LIPUS operates at an intensity of less than 3 W/cm^2^, which is significantly lower than conventional ultrasound. It is primarily used for non-invasive stimulation [91]. In contrast, high-intensity ultrasound exceeds this threshold and is commonly applied for tissue ablation or thermal applications. Recent studies have demonstrated that LIPUS stimulation can increase EV yield, making it a promising strategy for scalable and non-invasive EV biomanufacturing. LIPUS has been shown to significantly enhance EV yield across various cell types. Zhao et al. reported a ~ 1.5-fold increase in EV production when human ovarian cancer cell lines (A780 and SKOV3) were exposed to LIPUS at 0.5 W/cm^2^ for 60 min. It is suggested that LIPUS stimulation increases EV release by modulating the expression of genes associated with EVs, including CHMP2B, CHMP5, and YKT6 [76]. Similarly, Li et al. observed a 3.66-fold increase in EV secretion from BMSCs following LIPUS stimulation at 300 mW/cm^2^ for 15 min. LIPUS-treated BMSCs exhibited upregulated expression of Alix, HGS, YBX-1, LRP5 and MYC gene related EV biogenesis or cell cycle [92]. Enhanced cytoskeletal activity driven by LIPUS was proposed to play a role in the increased production of EVs. Deng et al. observed an approximately fivefold increase in EV yield following the stimulation of human astrocytes with ultrasound at an intensity of 280 mW/cm^2^ for 15 min [93]. High-intensity ultrasound has also demonstrated its capability to enhance EV production. Yamaguchi et al. irradiated ultrasound at 3.0 W/cm^2^ for 15 min to C2C12 cells, resulting in a twofold increase in EV yield. A transient rise in intracellular calcium levels was observed during stimulation, which was essential for EV production. In the absence of calcium, the increase in EV production was nullified. These findings indicate that calcium influx plays a critical role in the mechanism [94]. The cavitation property of ultrasound generates microbubbles that induce sonoporation. This process creates transient micro-pores in the cell membrane enhancing membrane permeability. As a result, it facilitates the intracellular delivery of external molecules and improves EV secretion. Li et al. demonstrated that sonoporation using 1.25 MHz ultrasound pulses (duration: 8 µs, 0.34 MPa) in RAW 264.7 cells led to a twofold increase in EV yield [95]. Similarly, Yuana et al. reported a twofold increase in EV yield when cells treated with microbubbles were exposed to 1.5 MHz ultrasound pulses (duration: 100 µs, 1 kPa) [96].

Both LIPUS and high-intensity ultrasound demonstrate significant potential to enhance EV production, albeit through different mechanisms. LIPUS offers a non-invasive approach that modulates EV-related gene expression and cytoskeletal activity, while high-intensity ultrasound leverages calcium influx and cavitation effects to stimulate EV release. The versatility and scalability of ultrasound-based EV production make it a promising strategy for advancing EV biomanufacturing and therapeutic applications.

### 3D culture-based structure modulation

Traditional 2D culture systems have been widely used for EV research, but they face major limitations in large-scale applications. In 2D culture, cells are restricted to the surface area of culture containers, which limits cell proliferation and makes high-density cell expansion difficult. In contrast, the 3D culture system enhances EV secretion by not only allowing for large-scale cell culture but also providing a dynamic microenvironment that better mimics the natural ECM. These 3D platforms promote cell-to-cell or cell-to-ECM interactions and increase signal transduction; both are crucial for EV secretion. Additionally, 3D culture systems sustain high cell densities and maintain long-term viability, making them an effective alternative for high-yield EV production. This section explores advancements in 3D culture techniques for large-scale EV production, focusing on spheroid, microparticle-based bioreactor, hollow-fiber bioreactor, and scaffold-based systems for therapeutic and industrial applications.

#### Spheroid

Spheroids are 3D cellular aggregates that self-assemble into spherical structures under non-adherent culture conditions. This self-assembly process facilitates the aggregation of adherent cells without the need for substrate attachment, resulting in spheroids with well-defined morphology. Spheroids exhibit a wide range of sizes, typically spanning from 50 to 1000 μm in diameter, depending on the cell type, culture conditions, and formation methods. Smaller spheroids (50–150 μm) are more favorable for oxygen and nutrient diffusion, maintaining viability throughout the structure. In contrast, larger spheroids (> 200 μm) often develop hypoxic cores and necrotic regions due to limited oxygen and nutrient penetration, better mimicking the conditions of solid tumors. These size-dependent characteristics make spheroids versatile models for studying diverse biological processes and disease mechanisms. Spheroids hold significant physiological importance as they closely mimic the *in vivo* tissue environment, making them indispensable tools in cancer research, drug screening, and tissue regeneration studies. Unlike traditional 2D culture systems, which often fail to replicate the complexity of living tissues, spheroids provide a three-dimensional architecture that facilitates enhanced physical and chemical cell–cell interactions, leading to more biologically relevant outcomes. The 3D microenvironment of spheroids allows for the establishment of oxygen and nutrient gradients, similar to those observed in solid tumors or native tissues. This gradient enables the study of cellular heterogeneity, including the behavior of hypoxic core regions, which are critical in understanding tumor resistance to therapies. For example, spheroids can emulate tumor microenvironments, providing a platform to investigate cancer invasion, metastasis, and response to anticancer agents under conditions that are more representative of clinical scenarios. Additionally, spheroids promote ECM deposition, which is essential for cell adhesion, migration, and differentiation. This aspect is particularly relevant in tissue engineering and regenerative medicine, as spheroids can mimic the structural and biochemical properties of native tissues. Spheroids also provide a physiologically relevant model for high-throughput drug screening, enabling the evaluation of drug efficacy and toxicity in a controlled yet biologically relevant environment. Their ability to mimic drug diffusion and retention within 3D structures offers insights into how therapeutic agents interact with tissues at a multicellular level. In immunology, spheroids are used to model immune-tumor interactions by co-culturing tumor cells with immune cells, allowing for the study of immune evasion mechanisms and the efficacy of immunotherapies. This approach has been instrumental in advancing our understanding of immune checkpoint inhibitors and T-cell activation within the tumor microenvironment.

The concept of cell aggregation, a key principle in spheroid culture, was first explored by Holt Freter in the 1940s during his studies on embryonic development and cell–cell interactions. However, the modern concept and application of spheroid culture as a 3D *in vitro* model were widely developed and popularized in the 1970s by Hamilton et al., particularly for studying tumor biology. Over the past decade, various techniques for spheroid culture have been developed, including the hanging drop method, low-adhesion plates, spinner flasks, and magnetic levitation. The hanging drop method involves placing cells on the lids of petri dishes, where they aggregate into spheroids; however, this approach is limited by challenges in medium exchange and scalability. Low-adhesion plates prevent cells from attaching to the substrate, thereby facilitating spheroid formation. Spinner flasks use agitation to promote spheroid formation, supporting larger-scale production. Magnetic levitation employs magnetic nanoparticles to drive the self-assembly of cells into spheroids, addressing necrosis-related limitations while also enabling imaging capabilities.

Many studies have also shown that 3D spheroid systems produced more EVs compared to conventional 2D culture methods. Kim et al., when they seeded MSCs at the same density resulted in a 6.7-fold higher yield of EVs from 3D spheroid culture compared to the traditional method. Miceli et al. [97]. compared the yield of the MSC-derived EVs between 2 and 3D spheroid cultures and found that there are increased EVs secreted in the 3D spheroid MSC compared to 2D culture MSC [98]. Also, Tu et al. isolated EVs from a pancreatic cancer cell line (PANC-1) grown in 3D spheroids. The spheroid 3D culture system produces a higher yield of EVs compared to 2D culture system. In addition, 3D spheroid with dynamic culture can improve released EVs [99]. Lim et al. have shown enhanced EV production from 3D MSC spheroid with dynamic culture condition compared to 2D MSC culture condition. Also, Yuan et al. found that 3D spheroid MSC-derived EVs in dynamic culture system have increased production compared to 2D MSCs-derived EVs [100]. Cha et al. amplified EV secretion from MSC with dynamic 3D-spheroid culture compared to the conventional culture method [101]. Also, Khan et al. cultured human choriocarcinoma JAr cells under traditional 2D monolayer culture and 3D spheroid suspension culture. The findings indicated that the 3D culture system was more active in secreting EVs, producing approximately 1.5 to 2.5 times higher yield compared to the 2D system [102]. Despite this increased yield, no significant differences were observed in terms of morphology and size between EVs derived from the two culture systems.

Several mechanisms have been proposed to explain the increased production of EVs in spheroids. First, hypoxia within the spheroid core has been identified as a key factor. The central regions of spheroids often experience oxygen deprivation due to limited oxygen diffusion. Hypoxia has been reported to promote EV secretion in monolayer cultures, and this effect is likely replicated in spheroid conditions. Second, changes in cell morphology and cytoskeletal structure are also implicated. In 3D cultures, MSCs undergo morphological changes, adopting a non-adherent, rounded shape. This is accompanied by reduced expression of F-actin and remodeling of the actin cytoskeleton, leading to decreased cytoskeletal tension. Notably, the depolymerization of F-actin is crucial for docking secretory vesicles at the fusion sites of the cell membrane, which may create a favorable environment for EV synthesis and release in 3D cultures. Third, enhanced cellular metabolic activity in spheroid cultures has also been suggested as a contributing factor to increased EV production. Lastly, the increased surface area of cells within spheroids may further facilitate the release of EVs. However, despite these observations, the precise mechanisms underlying the enhanced EV production in spheroids remain poorly understood. Moreover, the number of cells within a spheroid, the spheroid size, and the culture conditions can result in significant variability in EV yield, ranging from a twofold to a 100-fold increase. To achieve effective large-scale production of EVs, it is crucial to establish more standardized and optimized protocols tailored to spheroid cultures.

#### Microcarriers with bioreactor system

Microcarrier-based bioreactor systems represent an innovative and efficient approach for the large-scale cell culture and production of EVs. Microcarriers provide a large surface area for cell attachment and proliferation, while bioreactors enable precise control of environmental parameters such as temperature, pH, and oxygen levels, optimizing cell growth and EV secretion [103–106]. These systems are scalable, minimize contamination risks through automation and closed environments, and maintain the natural characteristics of cells in a 3D culture setting. However, challenges such as potential cell damage due to shear forces, difficulties in EV purification, and high initial costs must be addressed [107].

Numerous studies have demonstrated the effectiveness of microcarrier-based bioreactor systems in enhancing EV production yields. Fuzeta et al. developed a scalable method for producing EVs from bone marrow (BM), adipose tissue (AT), and umbilical cord matrix (UCM)-derived MSCs using animal product-free SoloHill plastic microcarriers (PALL). The vertical-wheel bioreactor (VWBR) system achieved a 5.7-fold increase in EV concentration and a threefold increase in EV productivity per cell compared to static T-flask systems [104]. Similarly, Haraszti et al. showed that microcarrier-based 3D culture using Star-Plus Microcarriers (SoloHill) and Wharton’s Jelly-derived MSCs produced 20-fold more EVs compared to 2D culture systems [105]. Additionally, Jalilian et al. demonstrated a 24-fold increase in EV concentration when culturing BM-MSCs in a microcarrier-based 3D culture system using Synthemax II microcarriers with Vertical-Wheel™ bioreactor compared to a 2D culture system [105]. Duan et al. showed that human synovial fluid MSCs (hSF-MSCs) cultured on Cytodex microcarriers (GE Healthcare) in a Rotary Cell Culture System (RCCS) for 3D dynamic culture produced approximately 1.5 times more EVs compared to traditional 2D flask methods [105]. Moreover, Huang et al. demonstrated that porous GelMA microcarriers, when combined with a herringbone-structured microfluidic chip, achieved a 21-fold higher EV yield compared to static flask culture [106].

This increase in EV productivity is likely due to several factors. Fluid flow within the bioreactor system stimulates EV secretion. Cell aggregate formation facilitated by the microcarrier system enhances the release of paracrine factors and microvesicles. Hypoxic conditions in the microcarrier-based bioreactor further promote EV production. These factors collectively result in higher concentrations, productivities, and purities of EVs. However, further research is needed to quantify their individual contributions.

#### Hollow fiber bioreactor

The hollow-fiber bioreactor system is a perfusion-based 3D cell culture technology that utilizes semi-permeable hollow fibers to create a structured environment, promoting cell–cell and cell–matrix interactions [108]. A hollow fiber bioreactor has been developed for large-scale cell culture, providing an efficient platform for EV production. In this system, cells are cultured in the extracapillary space, while the intrafiber lumen facilitates continuous media perfusion [109]. This design optimizes nutrient exchange and waste removal, enabling high cell densities and enhanced EV secretion. The high surface-to-volume ratio supports efficient cell proliferation, while the hollow fiber's selective permeability allows for the effective separation of EVs from cells and debris, simplifying downstream processing. Unlike traditional 2D systems that require several hours to harvest EVs, 3D systems allow EV harvesting in less than 30 min per day, enhancing labor efficiency [110]. Furthermore, bioreactors offer precise monitoring and control of culture parameters, providing a physiologically relevant environment that better mimics *in vivo* cell–cell interactions. Several studies have demonstrated the advantages of hollow-fiber bioreactors for EV production. Cao et al. reported a 19.4-fold increase in EV production when culturing hUC-MSCs in a hollow-fiber bioreactor (FiberCell System, C2011) over 55 days compared to 2D culture systems, attributing this to the bioreactor’s ability to sustain higher cell densities and enable continuous EV collection [110]. Similarly, Yan et al. observed a 7.5-fold increase in EV production using the FiberCell Systems (C2025) hollow-fiber bioreactor with hUC-MSCs compared to 2D systems [111]. Gobin et al. produced EVs from four hBM-MSC donors over 25 days using the FiberCell Systems (P3202) hollow-fiber bioreactor. While direct comparisons with 2D systems were not conducted, their system consistently yielded EVs with a quality of 2.18 × 10^9^ particles/mL, suggesting the feasibility of continuous, large-scale EV production [112]. Additionally, Watson et al. demonstrated a 40-fold increase in EV production with HEK293 cells cultured in a hollow-fiber system, which was attributed to the EV-rich conditioned medium produced in the extracapillary space [113]. Kink et al. showed that hBM-MSCs cultured in the Quantum hollow-fiber bioreactor (Terumo BCT) over 72 and 96 h achieved a 38-fold higher production (1.84 × 10^1^° ± 1.3 × 10^9^ particles/mL) compared to a 24-h flask-based culture system. The hollow-fiber bioreactor’s small unit (2.1 m^2^) provides a surface area equivalent to 280T-75 flasks, requires 14-fold less media, and supports a 25-fold higher cell density (2.5 × 10° ells/mL) compared to flask-based systems [114]. This efficient EV concentration significantly enhances production yields, making hollow-fiber bioreactors a scalable and efficient platform for large-scale EV production.

#### Scaffold

Scaffolds physically support during cell culture and transplantation by facilitating cell adhesion and survival. Scaffolds can be made from natural or synthetic materials with various physical properties that allow them to be customized for specific cellular environments [115]. Cells on all surfaces interact with surrounding cells, ECM, or polymers in the scaffold-based 3D culture system [116]. Multidirectional interaction in scaffolds enhances cell–cell, cell-ECM, or polymer communication, unlike the limited interactions in a single plane found in the 2D culture system [117]. It also allows cells to maintain their native morphology while promoting biochemical and mechanical signaling to increase cell function. Several research has shown that natural ECM-mimicking scaffolds enhance EV production [118, 119]. For instance, Titanium metallic biomaterials that mimic bone tissue ECM and hydrogel that is composed of natural or synthetic polymers. Hydrogel can be designed to replicate ECM properties like swell in aqueous environments. Yu et al. engineered a 3D culture model using collagen hydrogels and compared EV production between 3 and 2D conditions. The results demonstrated that the 3D culture model significantly increased EV yield, achieving a 2.5-fold enhancement in EV production efficiency. One key advantage of hydrogels is their tunable stiffness, which can directly influence EV production. By modulating stiffness, hydrogel-based scaffolds can enhance EV secretion [118]. Patwardhan et al. reported that stiff ECMs promote EVs secretion at 2.5 times higher levels compared to soft environments through a YAP/TAZ-pathway-dependent mechanism when MCF-7 and MDA-MB-231 human breast cancer cells were cultured on polyacrylamide (PA) hydrogels [119]. These findings highlight the critical role of matrix stiffness in optimizing EV yield, suggesting that tailored hydrogel properties can be strategically employed to maximize EV production.

3D printing is an advanced scaffold fabrication technique that allows for precise control over scaffold architecture. This additive manufacturing technology enables the creation of complex in situ environments that closely replicate physiological conditions [120]. Man et al. explored the application of 3D-printed titanium scaffolds for EV production, demonstrating that hBMMSCs cultured on these biomimetic scaffolds exhibited a 2.22-fold increase in EV yield compared to conventional 2D cultures. Interestingly, the pore geometry of the scaffold significantly influenced EV production. A triangular pore structure led to a 2.15-fold higher EV yield compared to a square pore structure [121]. Variations in porosity, permeability, and scaffold microarchitecture likely contributed to differences in cellular differentiation efficiency and EV secretion. These findings underscore the critical role of scaffold design in optimizing EV production and suggest that tailored 3D-printed scaffolds hold great promise for enhancing EV-based applications.

### Cell-derived nanovesicles (EV-mimic particles)

In addition to improving natural EV production, strategies are being developed to break down cell membranes and facilitate their reassembly into cell-derived nanovesicles (EV-mimetic nanovesicles, EV-MNVs). EV-MNVs are engineered nanoparticles designed to mimic the structural and functional characteristics of EVs. They consist of an EV-like membrane, which is composed of a lipid bilayer derived from cell membranes or artificially assembled phospholipids, and they typically exhibit a spherical shape ranging from 50 to 500 nm in size. Typically, their surface charge is negative, which can tuned by chemical modifications or compositional changes [122]. EV-MNVs are capable of effectively encapsulating and delivering bioactive molecules, including RNA, proteins, and drugs, mimicking the functional properties that are of natural EVs. Various fabrication approaches, including sonication, nitrogen cavitation, extrusion, and microfluidics, are utilized to develop these nanoparticles, which can also be tailored into hybrid versions by integrating synthetic components [122]. EV-MNVs not only preserve the biocompatibility and drug delivery efficiency of natural EVs but also enable large-scale and reproducible production with consistent size and controlled cargo composition. Furthermore, engineering strategies such as surface protein modification, optimized drug loading, enhanced targeting capabilities, immune response modulation, and controlled *in vivo* half-life can significantly improve their therapeutic efficacy [122].

The properties of EV-MNVs vary depending on the fabrication process. They can be produced either by disrupting cells physically or chemically to extract membranes, which are then reassembled into nanoparticles, or by isolating EVs directly from cells and further engineering them [123]. Isolating only the cell membrane to create empty nanoparticles enhances drug loading efficiency while minimizing immune reactions or adverse effects caused by endogenous biomolecules. On the other hand, when both the cell membrane and cytoplasmic components are retained during fabrication or when extracted EVs are reassembled, the bioactive components of natural EVs are preserved, enabling additional biological functionalities [122]. EV-MNVs hold significant potential for overcoming the limitations of conventional EVs in various fields, including disease treatment and gene delivery. With continued research and development, they are expected to evolve into more precise and effective nanovesicle platforms. In this section, EV-MNVs are presented as an alternative method for large-scale EV production. The discussion is focused solely on production yield, without addressing structural or functional modifications.

According to Wang L. et al., ultrasonication of hucMSCs for only 1 min produced EV-MNVs with 18.5-fold higher and a 100-fold faster production rate compared to natural EVs, while maintaining morphology, size distribution, and typical EV protein markers [124]. Gao et al. produced EV-MNVs from white blood cells using the nitrogen cavitation method. Nitrogen cavitation generates rapid pressure to create microbubbles, which break the cell membrane. The fragmented membranes self-assemble into EV-MNVs with 16 times higher yield than natural EVs [125, 126]. The serial extrusion technique has been utilized in a several studies to generate EV-MNVs at a yield up to 100–250 times higher than natural EVs with similar size and EV marker proteins [127–131]. This process involves sequentially passing cells through filters with decreasing pore sizes (e.g., 5, 1, and 0.2 μm), mechanically disrupting them into nanosized vesicles [132]. Microfluidic systems consist of micro-structures, allowing precise control of fluid flow offering simplicity and automation. As live cells pass through microchannels, they undergo mechanical disruption and self-assembly. Jo et al. demonstrated that EV-MNVs produced using both a flat system and silicon nitride microblades yielded more vesicles than naturally secreted EVs. Furthermore, slicing cells with microblades led to a 100-fold increase in vesicle yield [133, 134]. Hybrid EV-MNVs developed by combining EVs with synthetic liposomes maximize the benefits of both. This approach provides exogenous lipids for surface tunability, significantly enhancing yield. Jhan et al. demonstrated that serial extrusion of natural EVs with synthetic lipids through a ~ 100 nm membrane filter led to a 6-to 43-fold increase in vesicle yield.

## Conclusion

This review addressed several approaches to improving EV production, from chemical modulation of culture conditions to mechanical stimulation approaches and structural manipulation through artificial 3D culture systems, as well as the generation of cell-derived nanovesicles. These are promising strategies to circumvent the current limitations of EV yield, thus facilitating the large-scale use of EVs in biomedical research as well as their clinical application (Table 1).

**Table 1:** Summary of strategies for enhancing EV production

Strategy		Typical fold increase	Relative effectiveness	Scalability	Pros	Cons
Chemical modulation	Nutrient starvation	2.5–4.3 fold	Moderate	High	High purity Simple Cost-effective Applicable to diverse cell types	Risk of cell stress or apoptosis Risk of EV functionality and stability impairment
	Ph modification	2.5–69 fold	High	Moderate	Simple Cost-effective Rapid EV production Applicable to diverse cell types	Limited understanding of mechanisms Mainly studied in cancer cells Risk of EV functionality and stability impairment
	Temperature change	2–22 fold	High	Moderate	Simple Easy to control Non-invasive physical stimulus Applicable to diverse cell types	Limited understanding of mechanisms Risk of cell damage Risk of altered EV cargo
	Hypoxia	1.5–tenfold	Moderate	Moderate	Mechanistic clarity via HIF pathway Promotes angiogenic and regenerative EV cargo Applicable to diverse cell types	Mainly studied in cancer cells Limited understanding of mechanisms Risk of cell stress or apoptosis Risk of apoptotic body contamination
	Cholesterol	4.7–26 fold	Moderate	Moderate	Mechanistic clarity Applicable to senescent cells Easily tunable with small-molecule drugs	Conflicting results across cell types
	Oxidative stress	1.3–32 fold	High	Moderate	Simple Cost-effective Applicable to various types of stimuli	Risk of cell stress or apoptosis Risk of EV functionality and stability impairment
	Chemical compound	1.7–40 fold	High	Moderate	Simple Applicable to various chemical compounds	Risk of EV functionality and stability impairment
Mechanical modulation	Shear stress	1.9–6.6 fold	Moderate–High	Moderate	Non-invasive and physiologically relevant Scalable using bioreactors or orbital shakers	Variable response depending on cell type and intensity Risk of cell stress or apoptosis
	Irradiation	1.7–3.3 fold	Low	Low	Applicable to diverse cell types Generates stress-responsive therapeutic evs	Safety concerns Cytotoxicity Requires specialized equipment
	Ultrasound	1.5–3.5 fold	Moderate	Moderate	Non-invasive Cost-effective Compatible with standard equipment	Potential for thermal or mechanical cell damage
3D culture-based structural modulation	Spheroid	1.5–100 fold	High	Moderate	Mimics *in vivo* microenvironment Improves cell–cell and ECM interaction	Variable yield depending on spheroid size and conditions Risk of hypoxia-related heterogeneity
	Bioreactor	1.5–24 fold	High	High	Suitable for large-scale production Automated system	High initial cost Risk of cell damage Complex EV purification process
	Hollow fiber bioreactor	7.5–40 fold	High	High	Suitable for large-scale cell culture Continuous perfusion Minimizes media use	High initial cost Complex EV purification process
	Scaffold	2.2–2.5 fold	Moderate	Low	Improves ECM-like interaction Applicable to various biomaterials Design precision via 3D printing	Complexity in EV purification Limited standardization Limited scalability

This table summarizes the reported strategies to increase EV yield, including their typical fold increase, relative effectiveness, scalability, advantages, and limitations. Relative Effectiveness was classified as: High; ≥ fivefold increase or consistently high enhancement across multiple studies. Moderate; 2–fivefold increase or conditional effectiveness depending on cell type or condition. Low; < twofold increase or inconsistent effects reported. Scalability was assessed based on the potential for large-scale or GMP-compliant production: High; Cost-effective, controllable via culture conditions, or easily automated. Moderate; Feasible with additional equipment or optimization; may involve technical challenges. Low; Limited by cytotoxicity, complexity, or poor reproducibility, hindering industrial application

Recently, combining physical and chemical stimuli to enhance EV production has gained attention in both research and industry. Its synergistic effects are under active investigation. In particular, dynamic 3D culture systems are often used to apply both chemical and mechanical stimulation at the same time. These combined stimuli can improve cellular responses and lead to enhanced EV secretion. For example, MSCs were pretreated with TNF-α and IFN-γ and cultured as spheroids, EV production increased by about 1.5 times compared to untreated spheroids [97]. This result suggests that the combination of 3D structural modulation and chemical stimulation promoted higher cellular activity and EV release. Similarly, Lim et al. reported that adding TGF-β3 to MSC spheroid cultures increased EV production by about threefold. This increase was attributed to both structural modulation and the upregulation of the TGF-β signaling pathway [135]. In another study, Fuzeta et al. used a microcarrier-based bioreactor system under serum-free conditions. They suggested that the increase in EV production could be due to a synergy between nutrient starvation and the bioreactor environment. Furthermore, dynamic 3D culture systems expose cells to mechanical stresses—such as shear, tensile, and compressive forces—which are known to enhance EV production. However it is challenging to isolate and analyze the effects of individual stimuli in complex environments such as bioreactor systems where multiple mechanical stresses coexist [136]. Therefore, it is essential to quantify the relationship between mechanical stimulation and EV yield and to establish optimal culture conditions to maximize the industrial and clinical potential of EVs.

As EVs emerge as promising vehicles for diagnostics and treatment, high-level production at scale will be critical to driving their eventual clinical application. The scalability of EVs will greatly benefit the fields of regenerative medicine, targeted drug delivery, and disease biomarker discovery in terms of biological efficacy and stability.

Although many more studies revealed great progress in this area, significant barriers still persist, such as the heterogeneous nature of EVs, the high cost of large-scale production and the absence of standardized isolation and purification protocols. In addition, issues with EV stability, preservation of bioactivity, and possible immunogenicity should be overcome prior to broad clinical application.

Further studies are required to establish a low-cost, scalable production method for EVs, along with improved purification and modifications aimed at achieving cell-derived nanovesicles or bioengineered EVs to serve as prospects for clinical preferences. These approaches to advancements in bioreactor design, microfluidic-based isolation, and genetic engineering could help to improve overall EV yield without compromising functional integrity. Tackling these issues will be important for realizing the complete potential of EVs in the scientific and medical fields.

However, novel strategies to stimulate EV generation and support their use can be developed by combining principles in bioengineering, nanotechnology, and regenerative medicine. Addressing these challenges will be critical to unleashing the full power of EV-based therapeutics and diagnostics toward the realization of precision medicine.

## Data Availability

The datasets used and/or analyzed during the current study are available from the corresponding author on reasonable request.
